# Effects of the anti-diabetic imeglimin in hyperglycemic mice with septic shock

**DOI:** 10.1186/cc10628

**Published:** 2012-03-20

**Authors:** F Wagner, J Vogt, U Wachter, S Weber, B Stahl, M Groeger, O McCook, M Georgieff, P Fouqueray, T Kuhn, E Calzia, P Radermacher, E Fontaine, K Wagner

**Affiliations:** 1 University Medical School Ulm, Anesthesia, Ulm, Germany; 2 Poxel, Lyon, France; 3 Université Joseph Fourier, LBFA, Grenoble, France

## Introduction

Shock-related hyperglycemia impairs mitochondrial function and integrity [1], ultimately leading to apoptosis and organ failure [1,2]. Imeglimin is a new anti-diabetic drug with anti-hyperglycemic and anti-apoptotic properties [3]. Therefore we investigated its effects in hyperglycemic mice with septic shock.

## Methods

Immediately after cecal ligation and puncture, mice randomly received s.c. vehicle (*n *= 9) or imeglimin (*n *= 10; 100 μg/g). Fifteen hours later animals were anesthetized, mechanically ventilated and instrumented for a consecutive 6-hour observation period. After a second imeglimin bolus, colloid fluid resuscitation and continuous i.v. noradrenaline were titrated to maintain normotensive and hyperdynamic hemodynamics. Then 2 mg/g/hour glucose was infused to induce hyperglycemia. Glucose oxidation and gluconeogenesis were derived from blood ^13^C_6_-glucose and mixed expiratory ^13^CO_2_/^12^CO_2 _isotope enrichment during continuous isotope infusion. Liver mitochondrial activity was assessed using high-resolution respirometry [4,5], Bax, HO-1 and NF-κB expression by immunoblotting and EMSA. All data are median (quartiles).

## Results

Imeglimin decreased blood glucose levels (165 (153; 180) vs. 192 (184; 221) mg/dl, *P *= 0.007) by increasing whole body glucose oxidation (55 (52; 57) vs. 51 (49; 55)% of infused isotope, *P *= 0.085), which coincided with partial restoration of gluconeogenesis (0.38 (0.34; 0.41) vs. 0.31 (0.27; 0.33) mg/g/hour, *P *= 0.032), liver mitochondrial activity (oxidative phosphorylation (136 (134; 160) vs. 116 (97; 122) pmol O_2_/second/mg tissue, *P *= 0.003); maximal oxidative capacity (166 (154; 174) vs. 147 (130; 159) pmol O_2_/second/mg tissue, *P *= 0.064). Imeglimin increased liver HO-1, reduced liver Bax expression and attenuated NF-κB activation (all *P *< 0.001).

## Conclusion

Imeglimin improved whole body glucose utilization and gluconeogenesis, a well-established marker of liver metabolic capacity [4,5], and attenuated organ injury, at least in part due to inhibition of the mitochondrial apoptosis pathway.
